# Quick accomplishment and responsiveness were associated with a lower risk of mortality from cardiovascular disease among Japanese older men: the Japan Collaborative Cohort Study

**DOI:** 10.1265/ehpm.24-00323

**Published:** 2025-03-08

**Authors:** Miyu Moriwaki, Kokoro Shirai, Hironori Imano, Akiko Tamakoshi, Ryo Kawasaki, Hiroyasu Iso

**Affiliations:** 1 Division of Public Health, Department of Social Medicine, Osaka University Graduate School of Medicine, Suita, Osaka, Japan; 2 Department of Public Health, Kindai University Faculty of Medicine, Sayama, Osaka, Japan; 3 Department of Public Health, Faculty of Medicine, Hokkaido University, Sapporo, Hokkaido, Japan; 4 Institute for Global Health Policy Research, Bureau of International Health Cooperation, National Center for Global Health and Medicine, Shinjuku-ku, Tokyo, Japan

**Keywords:** Time management, Behavior, Cardiovascular disease, Cohort study

## Abstract

**Background:**

Quick accomplishment and responsiveness are behaviors related to time management by perceived control of time, such as a positive feeling of using one’s time well. In recent years, positive psychological states have been associated with a lower risk of cardiovascular disease (CVD). Thus, we investigated the associations of quick accomplishment and responsiveness with CVD mortality in a large cohort study.

**Methods:**

The study participants were 75,049 (30,901 men and 44,148 women) aged 40–79 between 1988 and 1990 and followed until the end of 2009. Hazard ratios (HRs) and 95% confidence intervals (CIs) of mortality from CVD according to quick accomplishment, responsiveness, and their combination were calculated after adjustment for potential confounding factors using the Cox proportional hazard model.

**Results:**

Quick accomplishment was associated with a lower risk of CVD mortality in women; a similar but marginally significant association was observed in men; the respective multivariable HR (95%CI) was 0.91 (0.83–0.99) and 0.93 (0.86–1.01). The presence of both quick accomplishment and responsiveness was associated with lower risk in men, which was confined to men aged 60–79; the respective multivariable HR (95%CI) was 0.88 (0.78–0.99) and 0.83 (0.72–0.96).

**Conclusions:**

Quick accomplishment was associated with a lower risk of CVD mortality. Quick accomplishment and responsiveness combined were inversely associated with CVD mortality risk among older men.

**Supplementary information:**

The online version contains supplementary material available at https://doi.org/10.1265/ehpm.24-00323.

## Introduction

Behavioral patterns such as quick accomplishment (a behavioral characteristic of trying to work efficiently) and responsiveness (a behavioral characteristic of making decisions quickly without procrastinating) are behaviors relating to time management. Time management consists of a behavior that aim to use the time while performing certain goal-directed activities effectively, and a behavior with a positive feeling of being able to use one’s time well [1]. While time management enhanced positive psychological states such as job satisfaction and well-being, it was associated with work-related stress, anxiety, emotional exhaustion, and depressive symptoms [2–7]. Some studies revealed these psychological states have been associated with the risk of cardiovascular disease [8–10]. However, no studies have investigated the associations of quick accomplishment and responsiveness with cardiovascular health. Thus, we investigated the associations of these behaviors with the risk of mortality from cardiovascular disease (CVD) in a large and long-term cohort.

Our *a priori* hypothesis is that quick accomplishment and responsiveness are inversely or positively associated with CVD mortality among middle-aged and older people.

## Methods

The Japan Collaborative Cohort (JACC) Study for Evaluation of Cancer Risks, sponsored by the Ministry of Education, Sport, Science, and Technology in Japan, was conducted from 1988 to 1990. The sampling methods and other details of the JACC Study have been described elsewhere [11, 12]. A total of 110,585 participants (46,395 men and 64,190 women), 40–79 years of age from 45 areas throughout Japan, were enrolled at baseline. Of them, 5,675 (2,488 men and 3,187 women) participants were excluded because they had a history of cancer or CVD at baseline. Then, 24,264 participants (10,754 men and 13,510 women) in 13 areas where the questionnaire did not include quick accomplishment and responsiveness were excluded. Finally, we excluded 5,597 (2,252 men and 3,345 women) participants with missing data for the psychological questionnaires about quick accomplishment and responsiveness from the analysis. Consequently, we used the data of 75,049 (30,901 men and 44,148 women) who lived in 32 communities across Japan. Informed consent was acquired before participants completed the questionnaires or from community leaders instead of individuals [11]. The ethics committees of Nagoya University School of Medicine (approval number: 177), Hokkaido University Graduate School of Medicine (approval number: 14-044), and the Osaka University Graduate School of Medicine (approval number: 14285-6) approved the present study.

In each study site, investigators conducted a standardized review of death certificates sent to each public health center, and mortality data were collected and centralized at the Ministry of Health and Welfare. The underlying causes of death were coded by the 10th Revision of the International Classification of Diseases and Related Health Problems (ICD-10). The follow-up of participants lasted until the end of 2009 or until death, whichever occurred first, except in four areas where the follow-up had ended in 1999, another four areas where follow-up ended in 2003, and two areas where follow-up ended in 2008. Follow-up endpoints included deaths from CVD as ICD-10 codes of I01 to I99.

The information about behavioral patterns such as quick accomplishment and responsiveness was obtained using a self-administered questionnaire. Quick accomplishment was defined as persons who answered “definitely yes” or “yes” to the question: “Are you in a hurry to achieve your jobs?” whereas those who answered “maybe yes” or “no” were without quick accomplishment. Responsiveness was defined as persons who answered “yes” to the question: “Do you have a quick response to things you need to deal with?” whereas those who answered “maybe yes” or “no” were without responsiveness. The Kappa coefficient for quick accomplishment and responsiveness was similar between men and women: 0.22 and 0.20, respectively.

We divided the participants into two groups to examine the association between either quick accomplishment or responsiveness and the risk of CVD mortality and four groups to explore the association between the combination of quick accomplishment and responsiveness and the risk.

The statistical analysis was based on age-adjusted incidence rates of CVD mortality during the follow-up period from 1988 to 2009. The sex-specific hazard ratios (HRs) and their 95% confidence intervals (CIs) of CVD mortality were calculated after adjustment for age and other potential confounding factors using the Cox proportional hazard models. These confounding variables included age (year), body mass index (BMI; <18.5, 18.5–24.9, and ≥25.0 kg/m^2^), history of hypertension (yes or no), history of diabetes (yes or no), smoking status (never, ex-smoker, or current), alcohol intake (never, ex-drinker, current drinker of 1–45, 46–68, or ≥69 g per day), participation in sports (rarely, 1–4, or ≥5 hours per week), walking frequency (rarely, 30–59, or ≥60 min per day), education level (elementary, junior high school or less, high school, or college or higher), employment status (employed, self-employed, housekeeping, unemployed, or others), perceived mental stress (low, moderate, high), purpose of life (“*ikigai*”) (yes, not particular, or no), perceived life enjoyment (yes, not particular, or no), and likely to be angry (yes, not particular, or no).

We also performed the age-specific analysis (age 40–59 and 60–79 years old, corresponding to the timing before and after retirement for employees in Japan at the baseline survey).

The statistical analysis was performed with the Statistical Analysis System (SAS) for Windows (version 9.4; SAS Inc, Cary, NC, USA). All P-values for statistical tests were two-tailed, and values of <0.10 and <0.05 were considered marginally and statistically significant.

## Results

Table 1 shows the sex-specific baseline characteristics of participants according to quick accomplishment and responsiveness. Participants with quick accomplishment and responsiveness compared with those without them were younger, more likely to be smokers, highly educated and employed, angry, had higher perceived mental stress, the purpose of life, and life enjoyment, while they were less likely to be hypertensive for men and women.

**Table 1:** Sex-specific baseline characteristics of participants according to quick accomplishment and responsiveness

	**Quick accomplishment**		**Responsiveness**	
	**−**	**+**	**−**	**+**
**Men**				
No. of participants	15,289	15,612	21,457	9,444
Age, years	58.1 (10.2)	56.0 (9.9)	57.6 (10.1)	55.7 (10.1)
Body mass index, kg/m^2^	22.7 (3.1)	22.7 (3.9)	22.6 (3.6)	23.0 (3.3)
History of hypertension	2,741 (17.9)	2,591 (16.6)	3,874 (18.1)	1,458 (15.4)
History of diabetes	854 (5.6)	907 (5.8)	1,227 (5.7)	534 (5.7)
Current smoker	7,663 (52.9)	8,095 (54.3)	8,300 (54.5)	4,330 (59.7)
Current drinker	10,903 (74.4)	11,601 (77.3)	15,611 (75.9)	6,893 (75.8)
Sports ≥5 h/week	1,073 (7.0)	1,092 (7.0)	1,408 (6.6)	757 (8.0)
Walking ≥1 h/day	6,931 (45.3)	7,174 (46.0)	9,912 (46.2)	4,193 (44.4)
College or more	2,333 (15.3)	2,731 (17.5)	3,160 (14.7)	1,904 (20.2)
Employment status				
Employed	5,145 (33.7)	6,524 (41.8)	7,958 (37.1)	3,711 (39.3)
Self-employed	5,152 (36.8)	5,294 (36.5)	7,112 (35.7)	3,334 (38.8)
House-keeping	15 (0.1)	9 (0.06)	16 (0.08)	8 (0.09)
Unemployed	2,743 (19.6)	1,930 (13.3)	3,543 (17.8)	1,130 (13.1)
Others	936 (6.7)	757 (5.2)	1,278 (6.4)	415 (4.8)
Perceive mental stress	2,020 (13.2)	4,811 (30.8)	4,177 (19.5)	2,654 (28.1)
Purpose of life	6,492 (42.5)	8,752 (56.1)	9,017 (42.0)	6,227 (65.9)
Perceive life enjoyment	5,051 (33.0)	6,665 (42.7)	6,859 (32.0)	4,857 (51.4)
Likely to be an angry	1,690 (11.1)	4,716 (30.2)	3,688 (17.2)	2,718 (28.8)
**Women**				
No. of participants	24,582	19,566	34,221	9,927
Age, years	58.0 (10.1)	56.4 (9.7)	57.7 (10.0)	56.1 (9.9)
Body mass index, kg/m^2^	23.1 (4.2)	22.8 (3.0)	22.9 (3.3)	23.3 (4.8)
History of hypertension	4,757 (19.4)	3,475 (17.8)	6,529 (19.1)	1,703 (17.2)
History of diabetes	859 (3.5)	605 (3.1)	1,142 (3.3)	322 (3.2)
Current smoker	985 (4.6)	1,010 (5.8)	1,277 (4.2)	718 (8.0)
Current drinker	4,934 (21.7)	4,789 (26.2)	7,045 (22.2)	2,678 (28.7)
Sports ≥5 h/week	965 (3.9)	875 (4.5)	1,332 (3.9)	508 (5.1)
Walking ≥1 h/day	11,094 (45.1)	9,311 (47.6)	15,917 (46.5)	4,488 (45.2)
College or more	2,139 (8.7)	1,970 (10.1)	2,851 (8.3)	1,258 (12.7)
Employment status				
Employed	4,869 (19.8)	5,357 (27.4)	7,804 (22.8)	2,422 (24.4)
Self-employed	3,734 (16.6)	3,459 (19.4)	5,327 (16.9)	1,866 (21.1)
House-keeping	7,458 (33.1)	5,053 (28.4)	9,786 (31.0)	2,725 (30.9)
Unemployed	5,238 (23.2)	2,976 (16.7)	6,812 (21.6)	1,402 (15.9)
Others	1,241 (5.5)	979 (5.5)	1,804 (5.7)	416 (4.7)
Perceive mental stress	3,110 (12.7)	5,443 (27.8)	6,138 (17.9)	2,415 (24.3)
Purpose of life	8,671 (35.3)	9,433 (48.2)	11,957 (34.9)	6,147 (61.9)
Perceive life enjoyment	7,854 (32.0)	7,874 (40.4)	10,627 (31.1)	5,101 (51.4)
Likely to be an angry	1,705 (6.9)	3,721 (19.0)	3,648 (10.7)	1,778 (17.9)

Age and body mass index are expressed as mean (standard deviation), and the others are expressed as numbers (percentages).

Supplemental Table 2 shows the sex-specific baseline characteristics of participants by the combination of quick accomplishment and responsiveness, and Supplemental Table 3 shows them stratified by age. Similar trends were observed between ages 40–59 and 60–79, as seen in Table 1 and Supplemental Table 1, respectively.

During 1,219,108 person-years of follow-up (median follow-up period 19.1 years), we documented 2,546 men and 2,403 women died from CVDs. Table 2 indicates the sex-specific HRs of CVD mortality according to quick accomplishment, responsiveness, and combination. After multivariable adjustment, quick accomplishment was associated with a lower risk of CVD mortality in women; a similar and marginally significant association was observed in men; the respective multivariable HR (95%CI) was 0.91 (0.83–0.99) and 0.93 (0.86–1.01). Responsiveness was not associated with the risk of CVD mortality in men and women. When examined by the combination of quick accomplishment and responsiveness, the presence of both quick accomplishment and responsiveness was associated with a lower risk of CVD mortality compared to their absence in men, a similar but not statistically significant association was observed in women. The multivariable HR (95%CI) of CVD mortality was 0.88 (0.78–0.99) and 0.92 (0.80–1.05), respectively.

**Table 2:** Sex-specific HRs of CVD mortality according to quick accomplishment, responsiveness, and combination

	**Quick accomplishment**		**Responsiveness**	
	**−**	**+**	**−**	**+**
**Men**				
No. at risk	15,289	15,612	21,457	9,444
Person-years	238,251	252,850	337,878	153,224
No. of cases	1,420	1,126	1,878	668
Age-adjusted HR (95%CI)	Ref.	0.90 (0.83–0.97)**	Ref.	0.91 (0.84–1.00)**
Multivariable HR (95%CI)^a^	Ref.	0.93 (0.86–1.01)*	Ref.	0.93 (0.84–1.02)

**Women**				
No. at risk	24,582	19,566	34,221	9,927
Person-years	399,089	328,917	561,257	166,749
No. of cases	1,495	908	1,927	476
Age-adjusted HR (95%CI)	Ref.	0.90 (0.83–0.98)**	Ref.	0.97 (0.81–1.15)
Multivariable HR (95%CI)^a^	Ref.	0.91 (0.83–0.99)**	Ref.	1.01 (0.91–1.13)


**Quick accomplishment**	**−**	**+**	**−**	**+**
**Responsiveness**	**−**	**−**	**+**	**+**

**Men**				
No. at risk	12,323	9,134	2,966	6,478
Person-years	190,827	147,051	47,425	105,799
No. of cases	1,186	692	234	434
Age-adjusted HR (95%CI)	Ref.	0.92 (0.84–1.01)*	0.95 (0.83–1.09)	0.85 (0.76–0.95)**
Multivariable HR (95%CI)^a^	Ref.	0.95 (0.86–1.04)	0.96 (0.83–1.11)	0.88 (0.78–0.99)**

**Women**				
No. at risk	21,140	13,081	3,442	6,485
Person-years	342,282	218,975	56,806	109,942
No. of cases	1,313	614	182	294
Age-adjusted HR (95%CI)	Ref.	0.91 (0.83–1.00)*	1.03 (0.88–1.20)	0.88 (0.77–1.00)**
Multivariable HR (95%CI)^a^	Ref.	0.92 (0.83–1.01)*	1.10 (0.93–1.29)	0.92 (0.80–1.05)

*p < 0.10, **p < 0.05

^a^Adjusted further for age, body mass index, smoking status, alcohol intake, education level, history of hypertension and diabetes, employment status, sports, walking, perceived stress, purpose of life, life enjoyment, and likely to be angry. HR: hazard ratio, CVD: cardiovascular disease, 95%CI: 95% confidence interval.

Table 3 indicates sex- and age-specific HRs of CVD mortality according to quick accomplishment, responsiveness, and combination. After multivariable adjustment, quick accomplishment was associated with a lower risk of CVD mortality in men and women aged 60–79 with marginally statistical significance; the respective multivariable HR (95%CI) was 0.92 (0.83–1.01) and 0.92 (0.84–1.01). Responsiveness was associated with a lower risk of CVD mortality in men aged 60–79 but not in those aged 40–59; the respective multivariable HR (95%CI) was 0.89 (0.79–0.99) and 1.02 (0.85–1.23). The presence of both quick accomplishment and responsiveness compared to their absence was also associated with a lower risk of CVD in men aged 60–79 but not in those aged 40–59; the respective multivariable HR (95%CI) was 0.83 (0.72–0.96) and 0.99 (0.78–1.26). Women did not have such an age difference in the association.

**Table 3:** Sex- and age-specific HRs of CVD mortality according to quick accomplishment, responsiveness, and combination

	**Quick accomplishment**		**Responsiveness**	
	**−**	**+**	**−**	**+**
**Men aged 40–59 years**				
No. at risk	8,062	9,738	11,882	5,918
Person-years	141,076	170,818	207,847	104,047
No. of cases	276	306	390	192
Multivariable HR (95%CI)^a^	Ref.	0.97 (0.81–1.15)	Ref.	1.02 (0.85–1.23)

**Men aged 60–79 years**				
No. at risk	7,227	5,874	9,575	3,526
Person-years	97,174	82,032	130,030	49,176
No. of cases	1,144	820	1,488	476
Multivariable HR (95%CI)^a^	Ref.	0.92 (0.83–1.01)*	Ref.	0.89 (0.79–0.99)**

**Women aged 40–59 years**				
No. at risk	13,272	12,012	19,112	6,172
Person-years	233,824	214,906	338,896	109,833
No. of cases	206	163	286	83
Multivariable HR (95%CI)^a^	Ref.	0.88 (0.71–1.09)	Ref.	0.95 (0.73–1.23)

**Women aged 60–79 years**				
No. at risk	11,310	7,554	15,109	3,755
Person-years	165,264	114,012	222,361	56,915
No. of cases	1,289	745	1,641	393
Multivariable HR (95%CI)^a^	Ref.	0.92 (0.84–1.01)*	Ref.	1.02 (0.91–1.15)


**Quick accomplishment**	**−**	**+**	**−**	**+**
**Responsiveness**	**−**	**−**	**+**	**+**

**Men aged 40–59 years**				
No. at risk	6,311	5,571	1,751	4,167
Person-years	110,314	97,533	30,762	73,285
No. of cases	217	173	59	133
Multivariable HR (95%CI)^a^	Ref.	0.98 (0.79–1.20)	1.05 (0.78–1.41)	0.99 (0.78–1.26)

**Men aged 60–79 years**				
No. at risk	6,012	3,563	1,215	2,311
Person-years	80,512	49,517	16,662	32,514
No. of cases	969	519	175	301
Multivariable HR (95%CI)^a^	Ref.	0.95 (0.85–1.06)	0.93 (0.79–1.10)	0.83 (0.72–0.96)**

**Women aged 40–59 years**				
No. at risk	11,193	7,919	2,079	4,093
Person-years	197,256	141,640	36,568	73,265
No. of cases	181	105	25	58
Multivariable HR (95%CI)^a^	Ref.	0.83 (0.64–1.06)	0.81 (0.53–1.24)	0.90 (0.66–1.24)
				
**Women aged 60–79 years**				
No. at risk	9,947	5,162	1,363	2,392
Person-years	145,026	77,334	20,238	36,677
No. of cases	1,132	509	157	236
Multivariable HR (95%CI)^a^	Ref.	0.94 (0.85–1.05)	1.16 (0.97–1.37)	0.92 (0.79–1.07)

*p < 0.10, **p < 0.05

^a^Adjusted further for age, body mass index, smoking status, alcohol intake, education level, history of hypertension and diabetes, employment status, sports, walking, perceived stress, purpose of life, life enjoyment, and likely to be angry. HR: hazard ratio, CVD: cardiovascular disease, 95%CI: 95% confidence interval.

## Discussion

In this large and long-term cohort of approximately 75,000 Japanese men and women aged 40–79, with over a million person-years, we found that quick accomplishment was associated with a lower risk of CVD mortality in women, and a similar but marginally association was observed in men. Quick accomplishment and responsiveness combined were associated with lower CVD mortality risk in men; that inverse association was confined to men ages 60–79.

The mechanisms behind why behavioral characteristics such as quick accomplishment and responsiveness are associated with a lower risk of CVD mortality are not fully understood. We suppose that quick accomplishment and responsiveness are the behavior with a positive feeling of being able to use one’s time well [1]. The mechanisms by which a positive psychological state may reduce the CVD mortality risk could be explained in two pathways [13, 14].

As for a biological pathway, positive psychological states may stimulate the activation of autonomic and neuroendocrine processes and reduce systematic inflammation [15, 16]. Blood concentrations of inflammatory markers such as C reactive protein and interleukin-6 were associated with an increased CVD risk [17, 18]. A previous study of 985 adults aged 25–74 residing in the United States showed that people with positive psychological states had lower levels of plasma C reactive protein and interleukin-6 [19].

As for a psychological pathway, a positive psychological state may be closely linked with healthy behaviors since it was postulated that positive moods or emotions may broaden the thought-action repertoires that increase personal resources ranging from physical and intellectual ones to social and psychological ones [20]. A population-based prospective study of 773 Dutch men aged 64 to 84 showed that men with high levels of optimism, one of the positive psychological states, were more likely to have health-promoting behaviors such as being physically active, avoiding smoking, and drinking moderately than men with lower levels of optimism [21]. In our study, people with quick accomplishment and responsiveness were likelier to be smokers and drinkers than those without them, probably due to their higher stress and social interaction, but they engaged more in sports.

In our study, quick accomplishment but not responsiveness was associated with a lower risk of CVD mortality in women. A possible explanation is that a positive psychological state may reflect quick accomplishment (a behavioral characteristic of trying to work efficiently) throughout the occupation categories. In contrast, responsiveness (a behavioral characteristic of making decisions quickly without procrastinating) could be reflected by a positive psychological state for the employed (23% of total women) but not for other occupation categories (77%) because many women are unlikely to have pressure by being employed.

No association between the presence of both quick accomplishment and responsiveness and the risk of CVD mortality among middle-aged men was not expected and warranted for discussion. Quick accomplishment and responsiveness may be standard psychological states for middle-aged people, and thus, their presence or absence is unlikely to influence the biological and psychological markers associated with CVD risk. On the other hand, the presence of both quick accomplishment and responsiveness among older people may reflect their well-being in general. Our study found positive correlations between quick accomplishment and responsiveness with mental stress, anger, purpose of life, and life enjoyment in both middle and older people. However, the adjustment for these factors did not change the associations materially. Residual confounding by misclassifying these variables and unmeasured variables is likely to exist. We speculate that the lower risk of CVD mortality in older men with both quick accomplishment and responsiveness could be due to higher social interaction and participation, which could avoid social isolation and loneliness, known as CVD risk factors for older people [22, 23]. Social network and participation were generally higher in women than men from earlier life and benefit more mental health in women than men [24], so no age differences in the associations were likely to be observed in women.

The strengths of this study were the enrollment of many participants from all over Japan and a long follow-up duration of a median of 19.1 years. This study had several limitations. First, the assessment of quick accomplishment and responsiveness was based on self-reporting, which was inevitable for misclassification. Second, the evaluation of quick accomplishment and responsiveness was performed only once during the baseline survey, and it is uncertain whether the changes during the follow-up affected the results. Third, the magnitude of association was generally weak, albeit statistically significant. However, this is an intrinsic issue for the epidemiological investigation of psychological factors and disease risk because of the misclassification of self-report exposure variables. Fourth, we could not exclude the possible influence of residual confounding, although we adjusted for potential cardiovascular risk factors. Last, a small number of deaths was observed in some reference groups among women, which made it underpowered to show potential associations.

In conclusion, quick accomplishment was associated with a lower risk of CVD mortality independent of conventional CVD risk factors. Quick accomplishment and responsiveness combined were inversely associated with CVD mortality risk among older men. Further studies are needed on the underlying pathophysiological mechanisms of quick accomplishment and responsiveness in the development of CVD.

## Supplementary information

**Additional file 1:** **Supplemental Table 1** Sex- and age-specific baseline characteristics of participants according to quick accomplishment and responsiveness. **Supplemental Table 2** Sex-specific baseline characteristics of participants by the combination of quick accomplishment and responsiveness. **Supplemental Table 3** Sex- and age-specific baseline characteristics of participants by the combination of quick accomplishment and responsiveness.

## Declarations

### Availability of data and material

De-identified data from this study are not available in a public archive. De-identified data from this study will be made available (as allowable according to institutional IRB standards) by emailing the corresponding author.

## References

[1] Claessens Brigitte JC, Eerde WV, Rutte CG, Roe RA. A review of the time management literature. Person Rev. 2007;36(2):255–76. doi: 10.1108/00483480710726136.

[2] Macan TH. Time management: test of a process model. J Appl Psychol. 1994;79(3):381–91. doi: 10.1037/0021-9010.79.3.381.

[3] Aeon B, Faber A, Panaccio A. Does time management work? A meta-analysis. PLoS One. 2021;16(1):e0245066. doi: 10.1371/journal.pone.0245066. 33428644 PMC7799745

[4] Kelly WE. No time to worry: The relationship between worry, time structure, and time management. Pers Individ Dif. 2003;35(5):1119–26. doi: 10.1016/S0191-8869(02)00322-7.

[5] Peeters MAG, Rutte CG. Time management behavior as a moderator for the job demand control interaction. J Occup Health Psychol. 2005;10(1):64–75. doi: 10.1037/1076-8998.10.1.64. 15656722

[6] Bond MJ, Feather NT. Some correlates of structure and purpose in the use of time. J Pers Soc Psychol. 1988;55(2):321–9. doi: 10.1037/0022-3514.55.2.321.

[7] Feather NT, Bond MJ. Time structure and purposeful activity among employed and unemployed university graduates. J Occup Psychol. 1983;56(3):241–54. doi: 10.1111/j.2044-8325.1983.tb00131.x.

[8] Kubzansky LD, Huffman JC, Boehm JK, Hernandez R, Kim ES, Koga HK, . Positive Psychological Well-Being and Cardiovascular Disease: JACC Health Promotion Series. J Am Coll Cardiol. 2018;72(12):1382–96. doi: 10.1016/j.jacc.2018.07.042. 30213332 PMC6289282

[9] Barth J, Schumacher M, Herrmann-Lingen C. Depression as a risk factor for mortality in patients with coronary heart disease: a meta-analysis. Psychosom Med. 2004;66(6):802–13. doi: 10.1097/01.psy.0000146332.53619.b2. 15564343

[10] Kubzansky LD, Kawachi I, Spiro A 3rd, Weiss ST, Vokonas PS, Sparrow D. Is worrying bad for your heart? A prospective study of worry and coronary heart disease in the Normative Aging Study. Circulation. 1997;95(4):818–24. doi: 10.1161/01.cir.95.4.818. 9054737

[11] Tamakoshi A, Ozasa K, Fujino Y, Suzuki K, Sakata K, Mori M, . Cohort profile of the Japan collaborative cohort study at final follow-up. J Epidemiol. 2013;23(3):227–32. doi: 10.2188/jea.je20120161. 23583921 PMC3700254

[12] Tamakoshi A, Yoshimura T, Inaba Y, Ito Y, Watanabe Y, Fukuda K, ; JACC Study Group. Profile of the JACC study. J Epidemiol. 2005;15(Suppl 1):S4–8. doi: 10.2188/jea.15.s4. 15881191 PMC8565873

[13] Rozanski A, Kubzansky LD. Psychologic functioning and physical health: a paradigm of flexibility. Psychosom Med. 2005;67(suppl 1):S47–53. doi: 10.1097/01.psy.0000164253.69550.49. 15953801

[14] Berkman LF, Blumenthal J, Burg M, Carney MR, Catellier D, Cowan JM, . Effects of treating depression and low perceived social support on clinical events after myocardial infarction: the Enhancing Recovery in Coronary Heart Disease Patients (ENRICHD) Randomized Trial. JAMA. 2003;289(23):3106–16. doi: 10.1001/jama.289.23.3106. 12813116

[15] Steptoe A, Gibson EL, Hamer M, Wardle J. Neuroendocrine and cardiovascular correlates of positive affect measured by ecological momentary assessment and by questionnaire. Psychoneuroendocrinology. 2007;32:56–64. doi: 10.1016/j.psyneuen.2006.10.001. 17157442

[16] Steptoe A, Wardle J, Marmot M. Positive affect and health-related neuroendocrine, cardiovascular, and inflammatory processes. Proc Natl Acad Sci USA. 2005;102(18):6508–12. doi: 10.1073/pnas.0409174102. 15840727 PMC1088362

[17] Harris TB, Ferrucci L, Tracy RP, Corti MC, Wacholder S, Ettinger WH Jr, . Associations of elevated interleukin-6 and C-reactive protein levels with mortality in the elderly. Am J Med. 1999;106:506–12. doi: 10.1016/S0002-9343(99)00066-2. 10335721

[18] Reuben DB, Cheh AI, Harris TB, Ferrucci L, Rowe JW, Tracy RP, . Peripheral blood markers of inflammation predict mortality and functional decline in high functioning community-dwelling older persons. J Am Geriatr Soc. 2002;50(4):638–44. doi: 10.1046/j.1532-5415.2002.50157.x. 11982663

[19] Zilioli S, Slatcher RB, Ong AD, Gruenewald TL. Purpose in life predicts allostatic load ten years later. J Psychosom Res. 2015;79(5):451–7. doi: 10.1016/j.jpsychores.2015.09.013. 26526322 PMC4684637

[20] Fredrickson BL. The role of positive emotions in positive psychology: The broaden-and-build theory of positive emotions. Am Psychol. 2001;56(3):218–26. doi: 10.1037/0003-066x.56.3.218. 11315248 PMC3122271

[21] Giltay EJ, Geleijnseb JM, Zitmana FG, Buijsse B, Kromhout D. Lifestyle and dietary correlates of dispositional optimism in men: the Zutphen Elderly Study. J Psychosom Res. 2007;63:483–90. doi: 10.1016/j.jpsychores.2007.07.014. 17980220

[22] Valtorta NK, Kanaan M, Gilbody S, Ronzi S, Hanratty B. Loneliness and social isolation as risk factors for coronary heart disease and stroke: systematic review and meta-analysis of longitudinal observational studies. Heart. 2016;102:1009–16. doi: 10.1136/heartjnl-2015-308790. 27091846 PMC4941172

[23] Freak-Poli R, Ryan J, Neumann JT, Tonkin A, Reid CM, Woods RL, . Social isolation, social support and loneliness as predictors of cardiovascular disease incidence and mortality. BMC Geriatr. 2021;21:711. doi: 10.1186/s12877-021-02602-2. 34922471 PMC8684069

[24] Shin H, Park C. Gender differences in social networks and physical and mental health: are social relationships more health protective in women than in men? Front Psychol. 2023;14:1216032. doi: 10.3389/fpsyg.2023.1216032. 38213610 PMC10782512

